# Open-Source Federated Learning Frameworks for IoT: A Comparative Review and Analysis

**DOI:** 10.3390/s21010167

**Published:** 2020-12-29

**Authors:** Ivan Kholod, Evgeny Yanaki, Dmitry Fomichev, Evgeniy Shalugin, Evgenia Novikova, Evgeny Filippov, Mats Nordlund

**Affiliations:** 1 Faculty of Computer Science and Technology, Saint Petersburg Electrotechnical University “LETI”, Saint Petersburg 197376, Russia; evgeny.yanaki@smartilizer.ru (E.Y.); dmitry.fomichev@smartilizer.ru (D.F.); evgenii.shalugin@smartilizer.ru (E.S.); esnovikova@etu.ru (E.N.); 2 Smartilizer Rus LLC, Saint Petersburg 197376, Russia; evgeny.filippov@smartilizer.ru; 3 AI Sweden, Zenseact AB, Smartilizer Scandinavia AB, 417 56 Goteborg, Sweden; Mats.Nordlund@smartilizer.com

**Keywords:** federated learning, Internet of Things, smart sensors, distributed learning, machine learning, deep learning, privacy

## Abstract

The rapid development of Internet of Things (IoT) systems has led to the problem of managing and analyzing the large volumes of data that they generate. Traditional approaches that involve collection of data from IoT devices into one centralized repository for further analysis are not always applicable due to the large amount of collected data, the use of communication channels with limited bandwidth, security and privacy requirements, etc. Federated learning (FL) is an emerging approach that allows one to analyze data directly on data sources and to federate the results of each analysis to yield a result as traditional centralized data processing. FL is being actively developed, and currently, there are several open-source frameworks that implement it. This article presents a comparative review and analysis of the existing open-source FL frameworks, including their applicability in IoT systems. The authors evaluated the following features of the frameworks: ease of use and deployment, development, analysis capabilities, accuracy, and performance. Three different data sets were used in the experiments—two signal data sets of different volumes and one image data set. To model low-power IoT devices, computing nodes with small resources were defined in the testbed. The research results revealed FL frameworks that could be applied in the IoT systems now, but with certain restrictions on their use.

## 1. Introduction

The Internet of Things (IoT) [1] combines a large number of smart devices (sensors, controllers, smartphones, etc.) that produce large volumes of data. The IoT technology serves as the basis for designing systems that control and manage large and complex systems, such as buildings, city infrastructures, critical infrastructure objects, manufacturing systems, and others.

The typical architecture of IoT [2,3] systems consists of different IoT devices connected with a large number of middle nodes. Often, the low-level devices are limited in their computational resources; therefore, data generated by them are collected and analyzed in a centralized resource using a powerful computational cluster. Usually, the data repository and the computational cluster are located in the Cloud [4].

The important drawback of collecting data in the centralized data warehouses is that this leads to an increase in total processing time, network traffic, and risk of unauthorized access to the data.

Another aspect of application of AI-based technologies relates to the security and privacy of the personal data being collected. This issue has become extremely important after adoption of several legislative regulations protecting personal data and privacy of citizens, such as the GDPR in European Union [5], CCPA [6] in the USA, and PDPA in Singapore [7]. They require transparent processing of personal data with an explicitly stated purpose and the consent of the data subject.

To solve these problems, Google proposed the paradigm of federated learning (FL) [8]. Its main idea is to build machine learning models based on data sets that exist across multiple data sources without exchanging data among the sources.

Currently, there is a number of surveys and reviews devoted to different aspects of implementing FL. For example, in [9,10], the authors present a detailed analysis of FL concepts and the architecture of FL systems, as well as privacy-aware issues, such as approaches to preserving data subject privacy and protecting against different types of attacks. Shokri et al. [11] investigated basic membership inference attacks that assume detection for a data record and black-box access to a model if this record was in the model’s training data set. In [12], the authors focused on poisoning attacks on federated multi-task learning and evaluated different attack scenarios, including alteration of raw data on target devices (direct attacks) and indirect modification of data on target devices using communication protocols.

In this survey, the authors investigated existing open-source FL frameworks:

- TensorFlow Federated (TFF) from Google Inc [13];
- Federated AI Technology Enabler (FATE) from Webank’s AI department [14];
- Paddle Federated Learning (PFL) from Baidu [15];
- PySyft from the open community OpenMined [16];
- Federated Learning and Differential Privacy (FL & DP) framework from Sherpa.AI [17].

Their capabilities, such as analysis accuracy and computational efficiency, were tested on three different data sets: the first two data sets contain signal time-series data, but they differ in their volumes, and the third one contains images.

In addition to the listed FL frameworks, there are also proprietary frameworks from leading IT companies. For example, NVIDIA added FL support to its NVIDIA Clara Train SDK starting with version 2.0. IBM is developing its own framework—IBM Federated Learning. These frameworks are briefly discussed in Section 4, but since they are proprietary, they were not evaluated by the authors.

To the best of the authors’ knowledge, there are not many surveys devoted to the analysis of the existing frameworks supporting FL. The most closely related survey is a comparative analysis of the FL frameworks in terms of privacy and communication architecture aspects [10]. Thus, the main contributions of this paper are as follows:

- Verification of the current capabilities of the analyzed open-source FL frameworks;
- Comparison of the built model’s accuracy;
- Comparison of the training process performance;
- Assessment of the ease of studying and deploying the FL frameworks;
- Analysis of the FL frameworks’ development and support states.

The paper is structured as follows. The key concepts of FL are given in Section 2. Section 3 outlines existing challenges and solutions in the considered area. Section 4 presents a brief description of existing open-source FL frameworks, describes the experimental setup, and provides the results of the evaluation of the selected frameworks. The paper ends with conclusions.

## 2. Federated Learning Concepts

**Figure 1:**
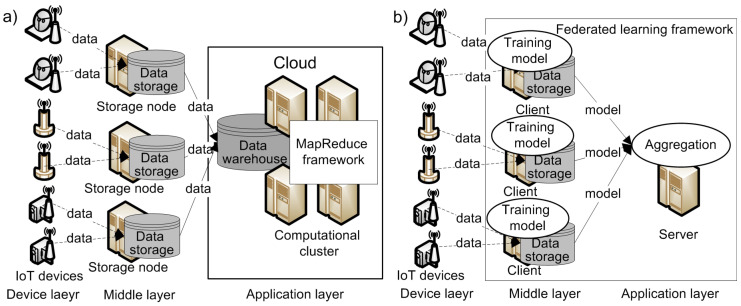
Internet of Things (IoT) system with: (**a**) distributed learning; (**b**) federated learning.

The typical architecture of an IoT system [2,3] used in many application domains consists of three layers of hierarchy (Figure 1):

- The device layer, which includes hardware devices that produce and collect data;
- The middle layer, which is responsible for transferring data from the device layer to the application layer for further processing;
- The application layer, which provides services or applications that integrate or analyze the data received from the middle layer.

The IoT system shown in Figure 1a receives data from different devices and collects them in the Cloud [4].

Currently, almost all large cloud service providers support machine learning environments for IoT systems. For example, Microsoft has proposed Azure Machine Learning [18], Google—the Cloud Machine Learning platform [19], Amazon—Amazon Machine Learning [20], and IBM—Watson Analytics [21]. These systems utilize the MapReduce programming model [22] for high-performance distributed computation. In this case, the middle layer of the IoT system connects IoT devices, such as sensors or cameras, to a Cloud. However, this usage scenario often results in an increase of the network traffic, the time of data processing, and the risk of an unauthorized access to the data, thus negatively affecting the overall performance of the data analysis process. FL can eliminate these disadvantages.

In 2016, McMahan et al. introduced FL as a distributed machine learning paradigm that supports data analysis, such neural network (NN) training, directly on the data storage; only the results of such processing, e.g., adjusted weights of the NN, are transmitted into the network to produce an aggregated analysis model [8]. Thus, unlike in traditional distributed systems, FL systems do not collect data in one data warehouse (Figure 1b).

There are three major components in an FL system (Figure 1b):

- Server (e.g., manager);
- Communication–computation framework;
- Clients (e.g., parties, data sources).

Formally, FL can be described with *N* clients, $c_{1},...,c_{N}$, which wish to train a machine learning model by consolidating their data sets, $d_{1},...,d_{N}$, respectively. A traditional distributed learning approach (Figure 1a) assumes collecting all $d_{i}$ data sets together and usage of *d* data set to train a model $m_{DL}$, where the *d* data set is defined as follows:

(1) $$d=d_{1}\cup \ldots \cup d_{N}.$$

In the FL paradigm, data from clients are processed collaboratively to train a model $m_{FL}$ in such a manner that any client $c_{i}$ does not expose its data $d_{i}$ to others. In addition, the accuracy of $m_{FL}$, denoted as $A(m_{FL})$, should be very close to the accuracy of a deep learning network $m_{DL}$, denoted as $A(m_{DL})$. Formally, let δ be a non-negative real number; then, the FL algorithm has δ-accuracy loss if:

(2) $$|A\left(m_{FL}\right)-A\left(m_{DL}\right)|<\delta .$$

Distributed learning and FL have some more differences (Table 1). First of all, the goal of distributed learning is to scale the parallel processing of a large amount of data, while the purpose of FL is to process data, i.e., train a model, directly on the data sources. Distributed learning works with identically and independently distributed (IID) data, which are collected in a single repository, from which they are extracted for further training. FL can treat IID data as non-IID data because training can be performed on sources connected with each other, i.e., sources that store different types of data about the same artifacts or events, or independent data sources. Another difference is the usage of network nodes. Distributed learning uses network nodes as computing resources for scaling, while FL uses nodes as data sources and performs calculations as close to the data as possible.

The FL process typically includes the following steps [9]:

- Identifying a problem to be solved;
- Modifying the client’s application (optional);
- Simulating prototyping (optional);
- Training the federated model;
- Evaluating the federated model;
- Deploying FL at the server and clients.

Thus, FL allows decreasing of:

- The risk of unauthorized data access, since data are not transmitted over the network;
- Network traffic because the training results are usually much smaller in volume than the data themselves;
- Time and cost of information transfer by reducing the amount of data transmitted;
- Requirements to the central computational cluster and the central storage, as there is no need to store all data in one place.

At the same time, to implement FL, the following challenges must be solved:

- Processing IID data as non-IID data, which can have different data partitions;
- Working with clients with different computing and storage capacity, as well as scale and stability;
- Implementation of different communication schemes: centralized and decentralized;
- Protection of transmitted analysis results from various types of attacks;
- Aggregation of the results obtained from data sources to calculate inequality (2).

## 3. Federated Learning Challenges

### 3.1. Data Partitioning

There are two different cases of how data are distributed in the IoT system [23]:

- Vertical partitioning, in which each storage node collects and stores data about different features of all objects;
- Horizontal partition, in which each storage node collects and stores data about all features of different objects.

Data set *d* from (1) is presented by a data matrix, where *z* refers to a number of samples and *p* defines a number of features:

$$d=\left(\begin{array}{ccccc} x_{11} & \ldots & x_{1k} & \ldots & x_{1p}\\ \ldots & \; & \ldots & \; & \ldots \\ x_{j1} & \ldots & x_{jk} & \ldots & x_{jp}\\ \ldots & \; & \ldots & \; & \ldots \\ x_{z1} & \ldots & x_{zk} & \ldots & x_{zp} \end{array}\right).$$

**Figure 2:**
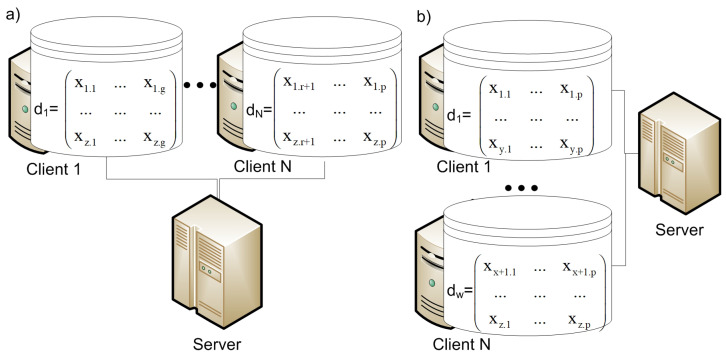
Data partitioning: (**a**) horizontal; (**b**) vertical.

Then, Figure 2 shows the vertical and horizontal data partitions for this data set.

Based on how data are distributed, FL systems can typically be categorized as horizontal or vertical FL systems [9,10,24].

### 3.2. Clients’ Settings

**Figure 3:**
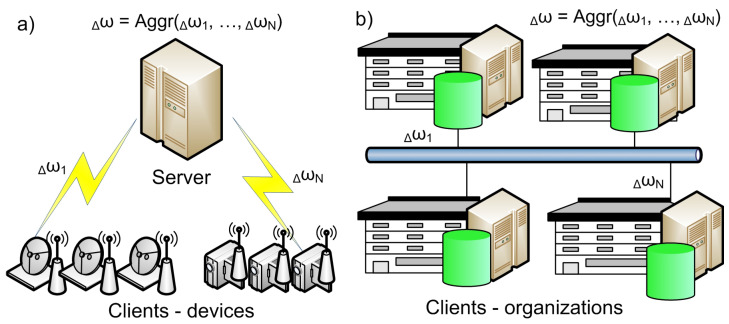
Types of federated learning (FL) systems: (**a**) cross-device; (**b**) cross-silo.

There are two different types of FL systems depending on the scale of federation [9,10] (Figure 3):

- Cross-silo systems have low scalable federation. They include organizations or data centers. Their numbers are small and rarely change;
- Cross-device systems have a scalable number of clients. They can be added and disabled at any moment of time. These are usually mobile devices and IoT devices.

Table 2 shows the differences between these two types of FL systems.

### 3.3. Communication Schemes

FL systems can implement two communication schemes between their components—centralized and decentralized [9,10].

The centralized scheme includes a central server. It is used to orchestrate different steps of the FL process and coordinate all clients therein. This scheme is typical for cross-device systems (Figure 3a). Since all selected nodes must send updates to a single entity, the server may become a bottleneck of the system.

In the decentralized scheme, all clients can coordinate themselves to obtain the global model. This scheme is often used in cross-silo systems where clients have high-performance resources (Figure 3b). Nevertheless, the specific network topology may affect the performance of the learning process.

### 3.4. Data Privacy and Security Mechanisms

Data privacy and security are essential properties of FL. There is a need to secure the models and the analysis process to provide meaningful privacy guarantees. The attacks can be performed at all stages of the FL process and can target all FL elements [9,10,24]:

- Training data set;
- Trained model;
- Client, and
- Server.

It is possible to outline two main types of FL-specific attacks—poisoning and inference attacks [9]. The first type of attack aims to modify either the input data set or the parameters of the trained model in order to bias it in a way that is preferable to the adversary. The goal of inference attacks is to get access to personal or confidential data. Depending on the attack implementation mechanisms, it is possible to derive information about the properties of training data or the labels of training samples, or to determine if the sample was used in the training process.

To protect FL against these attacks, the following security mechanisms are suggested:

- Secure multi-party computation (MPC) is a family of cryptographic protocols that allow a set of users to perform computations that use some private inputs without revealing them to other participants of the protocol [25]. The most widely used implementations of MPC are the ABY3 [26] and SecureML [27] protocols. These protocols implement a server–aided computational model in which data owners send their data in encrypted format to a number of servers, usually two or three, which perform model training or apply a pre-trained model to analyze input data.
- The Secure Aggregation (SecAgg) protocol proposed by K. A. Bonawitz et al. is another class of MPC-based technique used to secure the privacy of neural network training. However, in contrast to the ABY3 and SecureML protocols, the data owners participate in the training process by performing the training process locally and sending encrypted model weights to the aggregator, which implements the aggregation of the network gradients [28].
- Homomorphic encryption (HE) is a form of encryption that allows application of some mathematical operations directly on ciphertexts without decrypting them; the produced output is also encrypted, and after decryption, it corresponds to the result of the mathematical operations performed on the corresponding plain texts [29]. Currently, there are only a few implementations of fully homomorphic encryption, and all of them require significant research to increase speed performance. That is why, in major cases, partially homomorphic encryption protocols based on the RSA or Paillier encryption schemes are used as a compound part of security mechanisms [30].
- Differential privacy (DP) mechanisms preserve the privacy of data by adding noise to input data in order to mask the impact of the particular sample on the output. The privacy loss parameter defines how much noise is added and how much information about a sample could be revealed from the output, and the application of these mechanisms requires finding a balance between privacy loss and accuracy of the federated analysis model [31]. Different versions of differentially private algorithms for data classification [31,32,33] and clustering [34,35] are suggested.
- The trusted execution environment (TEE), e.g. Intel SGZ processors, guarantees the protection of the code and data loaded inside. This computational environment can be used inside the central server to increase its credibility [36].

### 3.5. Aggregation Algorithms

One of the key issues of FL is aggregating model changes made by clients into a single model, as the aggregation function should not impair the accuracy of the model (2).

The aggregation function depends on the model built in the FL process. For example, for centroid clusters constructed by the K-means algorithm, a prefix frequency filtering (PFF) method for data aggregation is suggested [37]. To support energy and computationally efficient data aggregation of similar data sets, the authors applied the K-means clustering algorithm on the data and then aggregated the generated clusters using PFF.

The ordered weighted averaging (OWA) [38] algorithm was one of the first proposed for aggregating the weight coefficients of NNs. The aggregation of the parameters is based on the amount of data in every node. When calculating a regular average, each data point has an equal “weight”, i.e., it contributes equally to the final value. Weighted averages, on the other hand, weight each data point differently. Yager and Filev suggested a generalization of the OWA operator called the induced ordered weighted averaging (IOWA) operator [39]. This operator firstly induces the ordering of arguments before their aggregation.

Currently, the popular aggregation function is the federated averaging algorithm (FedAvg) described in [8]. It averages the parameters of local models element-wise with weights proportional to the sizes of client data sets. There are variations of this algorithm. For example, the federated proximal algorithm (FedProx) [40] adds a proximal term for client local cost functions, which limits the impact of local updates by restricting them to be close to the global model. Agnostic federated learning (AFL) [41] is another variant of FedAvg; it optimizes a centralized distribution that is formed by a mixture of the client distributions.

One shortcoming of the FedAvg algorithm is that coordinate-wise averaging of weights may have a drastic detrimental effect on the performance, and hence hinders the communication efficiency. This issue arises due to the permutation-invariant nature of the NN parameters, i.e., for any given NN, there are many variations of it that only differ in the ordering of parameters and constitute local optimums, which are practically equivalent. Probabilistic federated neural matching (PFNM) [42] addresses this problem by finding permutations of the parameters of the NNs before averaging them. PFNM further utilizes Bayesian nonparametric machinery to adapt the global model size to the heterogeneity of the data. As a result, PFNM has better performance and communication efficiency; however, it was only developed for fully connected NNs and was tested on simple architectures.

In [43], federated matched averaging (FedMA) is proposed. It is a new layer-wise FL algorithm for modern convolutional neural networks (CNNs) and long short-term memory networks (LSTMs) utilizing the matching and model size adaptation underpinnings of PFNM.

## 4. Open-Source Federated Learning Frameworks

In this paper, the authors review five open-source FL frameworks that are currently under active development. They are TFF [13], FATE [14], PFL [15], PySyft [16], and FL & DP [17]. The following framework features were evaluated:

- Installation in simulation mode (on one computer) and in federated mode (on several nodes);
- Training of all machine learning models documented in the framework on all data sets using all available deep learning frameworks;
- Training time and system performance;
- Accuracy of trained models on test data using the methods available in the frameworks;
- Ease of use and study;
- Quality and availability of documentation;
- Development intensity;
- The framework support and development community.

In subsections below, these FL frameworks are briefly described. The authors also consider other frameworks briefly, but they are not included in the evaluation process, since they are not open-source projects.

### 4.1. TensorFlow Federated Framework

TFF is an open-source framework for deep learning on decentralized data [13]. The last version (0.17.0) of TFF uses the TensorFlow (TF) of version 2.3 for learning, estimation, and use of NNs. However, it does not support the use of GPUs. It is distributed under the Apache 2.0 license.

TFF implements base classes for the FedAvg and federated stochastic gradient descent (FedSGD) algorithms, a simple implementation of federated evaluation, and federated personalization evaluation. They can use several functions for aggregating the client model updates on the server:

- “Sum”, which sums values located at clients and outputs the sum located at the server;
- “Mean” computes a weighted mean of values located at clients and outputs the mean located at the server;
- “Differentially private” aggregates values located at clients in a privacy-preserving manner based on the differential privacy (DP) algorithm and outputs the result located at the server.

To create novel federated algorithms, TFF supports a core API. It is composed of classes, which define templates for stateful processes: aggregation of values, computation of estimates, and production of metrics. An analyst may develop their own analytical processes using them.

**Figure 4:**
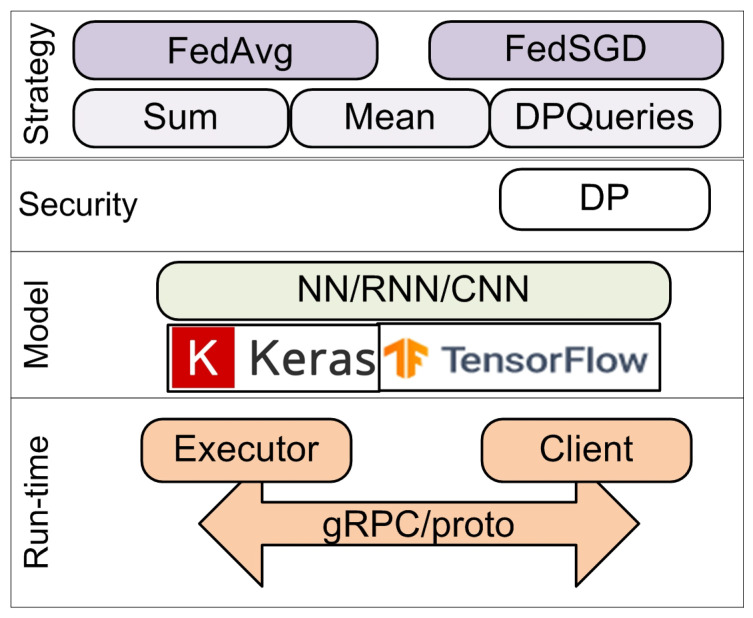
Architecture of TensorFlow Federated (TFF).

The architecture of TFF is shown in Figure 4. The current version of TFF implements only simulation mode. It is possible to use TFF via Google Colaboratory in a manner similar to TF. To use TFF locally, it is necessary to install the TFF package using Python’s pip package manager.

In addition, TFF includes classes for implementing a federated mode in the future. There are two possible deployment options in this mode:

- Native backend, which implements its own interaction between executors and clients based on gRPC technology and proto-object exchange;
- Non-native backend, which uses third-party distributed computing tools, such as the Hadoop cluster and MapReduce programming model.

The current version of TFF 0.17.0 is not complete and still lacks some important features required for practical application of the framework:

- The federated mode of operation is not implemented;
- Vertical and hybrid data splitting is not supported;
- The decentralized architecture of building the system is not supported;
- Only a differential privacy mechanism is used.

All these limitations prevent the use of TFF in commercial software development. However, it allows simulation of FL and selection of the optimal NN structure suitable for a user’s tasks. The TFF developers promise to release a fully functional framework in the near future. However, this can lead to significant changes in the TFF core and simulation mode implementations, which will require re-definition of the NN structure. It is also necessary to note the rapid development of TFF: five versions (versions 0.14.0, 0.15.0, 0.16.0, 0.16.1, and 0.17.0) were realized from July to November 2020; however, there is still no stable release version (1.x.x). The framework is supported by a quite big community with more than 60 contributors as of the end of October 2020.

### 4.2. Federated AI Technology Enabler Framework

FATE is an open-source project initiated by Webank’s AI Department to provide a secure computational framework supporting a federated AI ecosystem [14]. It is also distributed under the Apache 2.0 license.

FATE uses TF version 1.4 for deep learning models; however, currently, it is possible to use only the Dense layers of the NN. Interestingly, the official FATE documentation states that PyTorch can be used for deep learning; however, the authors could not find any code in the documentation. In addition to the NN, it also implements regression models (linear, logistic, and Poisson) and a decision tree (gradient-boosting decision tree).

FATE provides various implementations of the FL strategies (algorithms in terms of FATE) for each machine learning (ML) model and data partition. FATE supports analysis of horizontal (homogeneous) and vertical (heterogeneous) data partitions. The NN and logistic regression (Logs) with a horizontal data partition can be constructed either by the FedAvg [9] or secure aggregation (SecAgg) algorithms [28]. The latter algorithm is also used to build a gradient-boosting decision tree (GBDT) for horizontal data partitioning.

With vertical data partitioning, FATE trains the NN model in two steps: It uses encryption for all ML models: NN, Poisson regression (PR), logistic regression (LogR), and linear regression (LR). The SecureBoost algorithm is used to build the GDBT. Additionally, it implements a K-means clustering algorithm for vertical data partitioning. All strategies and models are implemented in the federatedml package.

To ensure security, FATE supports various implementations of secure MPC protocols and encryption methods: SecretShare MPC Protocol (SPDZ), RSA, Paillier, homomorphic encryption, and others. The Diffie–Hellman key is used in secure aggregation.

FATE implements only a centralized scheme, which includes:

- A server (arbiter) providing model aggregation and training management;
- A scheduler responsible for scheduling the FL process;
- An executer that implements the FL algorithm.

All of these components are included in the fate_flow package. For interaction, FATE uses gRPC technology with a data format in the form of proto-objects.

**Figure 5:**
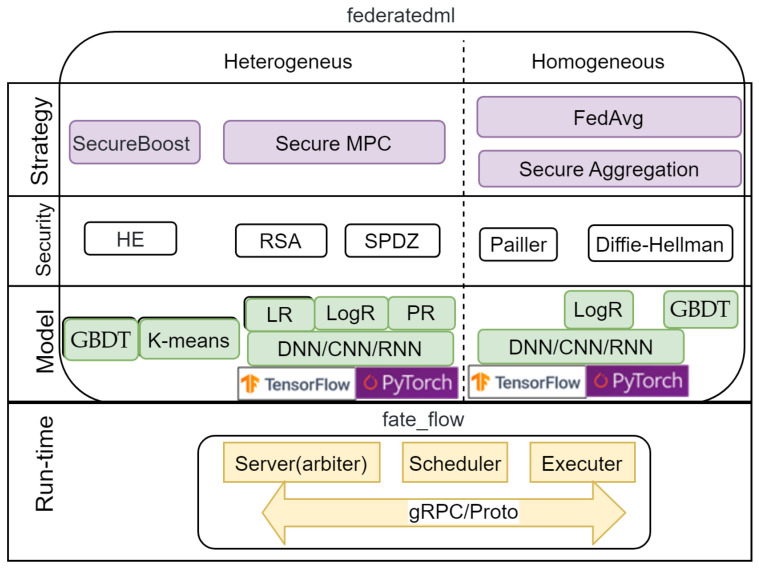
Architecture of Federated AI Technology Enabler (FATE).

The overview of FATE’s main components is shown in Figure 5.

FATE can be deployed in both simulation mode (standalone deployment) and federated mode (cluster deployment). The installation can be done manually by porting packages to the operating system, but it is recommended to use Docker containers to simplify the process. To deploy the framework, 6 GB of RAM and 100 GB of disk space are required on the server and client. Such requirements do not allow consideration of FATE as a cross-device framework for deploying FL in an IoT environment.

It should be also noted that FATE version 1.4.2 does not have built-in image readers for processing images.

Unlike TFF, FATE has a release version and has all the necessary features to be used in production. FATE does not currently support the decentralized architecture. However, it has good support from the developers’ community (60 contributors) and is being actively developed. Four versions of FATE were released in the last four months.

According to the authors’ opinion, one of serious shortcomings of FATE is the lack of a core API; this means that developers must modify the source code of FATE to implement their own FL algorithms.

### 4.3. Paddle Federated Learning Framework

PFL is an open-source FL framework with an Apache 2.0 license. It uses PaddlePaddle, which is a deep learning (DL) platform [15]. PFL can process horizontally and vertically partitioned data. Algorithms for each type of data partitioning are implemented in separate packages. The paddle_fl package should be used for horizontal data partition processing, and the mpc package for vertical data partition processing.

The paddle_fl package includes the NN and LR models. PFL implements FedAvg [9], SecAgg [44], and differentially private stochastic gradient descent (DPSGD) strategies to build them. DPSGD is a differentially private mechanism for securing data privacy. Horizontal data partition processing is performed in a centralized scheme. It includes the following elements:

- A server for model aggregation;
- A scheduler for planning the training process;
- A worker (trainer) for data processing and model building.

The ZeroMQ asynchronous messaging protocol is used to communicate between the server and workers.

The mpc package in PFL version 1.1.0 implements only the ABY3 protocol [26], which allows data processing on three computing party (CP) nodes. These nodes are peer-to-peer ones, and in this sense, PFL implements a decentralized scheme. The data owners or input parties (IP) transmit data to the CP in encrypted form using a secret sharing scheme. In general, the number of IPs can be more than three, but for the FL implementation, the number of IPs and CPs must be the same. The mpc package includes two models: NN and LR. After their training, they are transferred to the result party (RP). To detect data set intersection in vertically partitioned data, the private set intersection (PSI) technique is used [45].

PFL supports both simulation and federated modes, and it is recommended to use Docker containers for both deployment modes. To operate normally, PFL requires at least 6 GB RAM and at least 100 GB of HDD space. These requirements limit the usage of PFL in IoT systems.

**Figure 6:**
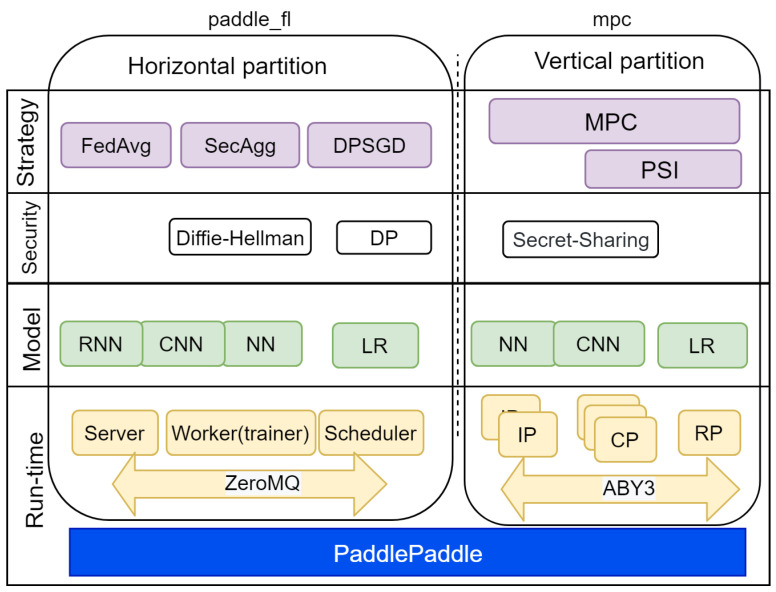
Architecture of Paddle Federated Learning (PFL).

The main components of the PFL are shown in Figure 6.

PFL supports two types of FL schemes—centralized and decentralized ones. PFL has a release version; thus, it has all features required to apply it in production. It is possible to conclude that this framework possesses the highest level of readiness for use in practice; however, it is fairly difficult to use it because it uses a little-known DL platform, has poor documentation, and has a small community—only 12 contributors.

### 4.4. PySyft Framework

In fact, PySyft is a open-source Python project for secure and private deep learning with an MIT License. It is a part of OpenMined ecosystem, which also includes the following projects:

- PyGrid is a peer-to-peer network of data owners and data scientists who can collectively train analysis models using PySyft;
- KotlinSyft is a project to train and inference PySyft models on Android;
- SwiftSyft is a project to train and inference PySyft models on iOS;
- Syft.js provides a web interface for the components listed above.

**Figure 7:**
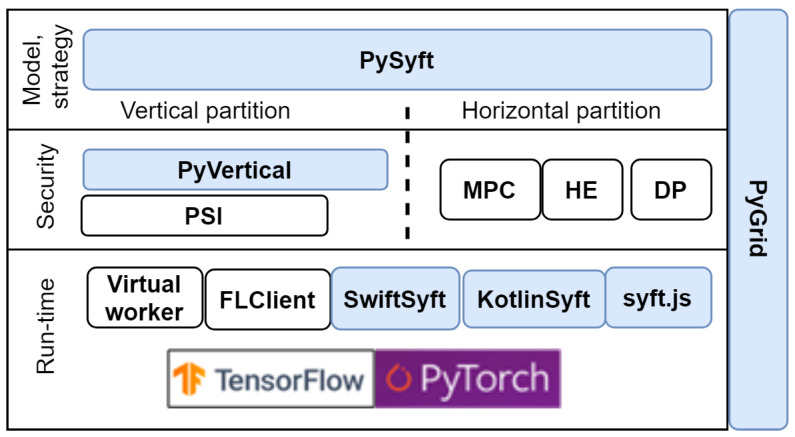
Architecture of PySyft.

PySyft simply decouples the private data from model training using the principles of FL secured with different privacy enhancing mechanisms. The PySyft architecture is shown in Figure 7.

The official PySyft documentation states that it supports the PyTorch and TF libraries; however, according to the developers’ forum and issues, the support of these libraries has only been planned, and has not yet been implemented.

The developers recommend using PySyft in a virtual environment with the Anaconda package manager. To simplify the installation and set-up process, it is possible to use a Docker container.

As a standalone library, PySyft functions only in simulation mode; to support federated mode, it has to be integrated with other projects of the OpenMined ecosystem, including PyGrid. However, currently, these projects like PySyft are under active development and do not have a release version. Thus, deploying PySyft in federated mode is impossible.

To process vertically partitioned data, the PyVertical project is included in the ecosystem; it is also under development.

Considering the fact that PySyft could be used only for testing and setting up the NN structure in simulation mode, it is possible to conclude that PySyft is not ready to be used in industrial products.

However, it should be noted that this framework has the largest community of contributors—over 250 developers—so it could be expected to have rapid development.

### 4.5. Federated Learning and Differential Privacy Framework

The FL & DP framework is a very simple open-source framework for FL, which is distributed under the Apache 2.0 license. The framework is developed by Sherpa.AI in collaboration with the Andalusian Research Institute in Data Science and Computational Intelligence research group from the University of Granada. It integrates TensorFlow version 2.2 for deep learning and the SciKit-Learn library for training linear models and clustering. This framework implements several aggregation algorithms for different models:

- A FedAvg aggregator for NN and LR;
- A weighted FedAverage aggregator and IOWA FedAverage aggregator for LR;
- A cluster FedAverage aggregator for centroid cluster models.

The framework implements the following privacy mechanisms to protect personal data:

- A simple mechanism adding random noise to binary data [46];
- An adaptive differential privacy mechanism based on Privacy Filters [47].

**Figure 8:**
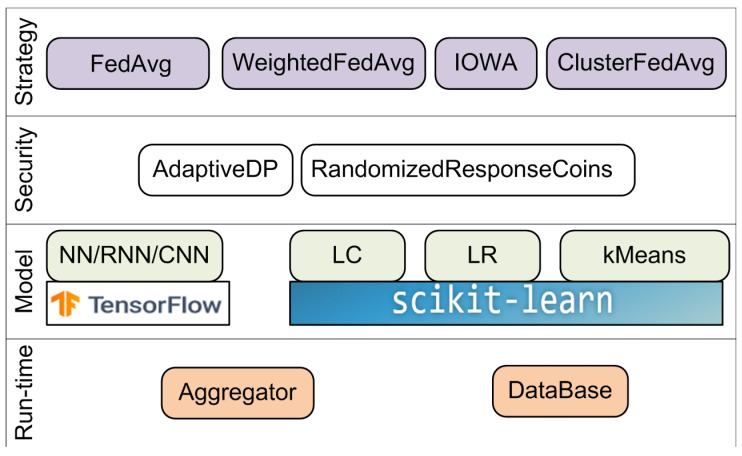
Architecture of the Federated Learning and Differential Privacy (FL & DP) framework.

The Federated Government module manages the learning process and implements a pipeline for each model. Figure 8 shows the main components of the FL & DP framework.

To install FL & DP, it is only required to import a set of packages without any complicated settings.

This framework does not have a release version, and it processes horizontally partitioned data in simulation mode only. Unlike TFF, it provides more privacy-preserving mechanisms, machine learning algorithms, and aggregation functions, but it also could not be applied to solve industrial tasks. Unfortunately, FL & DP has a low number of contributors—only six—and there is no detailed documentation on its interfaces or framework architecture.

Table 3 shows the summary of the open-source FL frameworks’ features.

### 4.6. Proprietary Federated Learning Frameworks

FL technologies are not only implemented in the form of open-source frameworks. Some leading companies have developed their own libraries, which are not open source and are distributed with a limited license.

A prime example of such a library is the NVIDIA Clara Train software development kit (SDK) [48], which has included FL since version 2.0. The current version of the framework that works in federated mode is 3.1. The NVIDIA Clara Train SDK does not open all source code of the framework and distributes it under its own license, which restricts its usage. The NVIDIA Clara Train SDK executes FL on a GPU only and it requires the support of CUDA 6.0 or higher. It uses TensorFlow and supports AutoML, which make development of models easy and familiar to developers. It implements a centralized workflow with the clients and server for managing the model training progress and transferring the original model to all clients. The solution is aimed primarily at the analysis of medical images. It has some built-in solutions for medical problems with the pre-trained models, which are provided as Model Applications and packaged as the Medical Model ARchive (MMARS) and the prepared environment. It also provides many built-in privacy mechanisms, such as the DPSGD, sparse vector technique, and percentile protocol. NVIDIA provides the Python-based Clara Train API for developers to improve their solutions. Despite the fact that NVIDIA Clara is not open source, it provides the possibility to improve the framework with the help of a bring-your-own-components (BYOC) method.

Another example is the IBM framework written in Python—IBM Federated Learning (IBM FL) [49]. IBM does not open the source code of the framework and distributes it under its own license, which restricts its use. The current version of the framework is 1.0.3, which works in both simulation and federated modes. A distinctive feature of the framework is the large number of implemented ML algorithms. In addition to NN, linear classification, and decision trees (ID3 algorithm), it includes support for the K-means, naive Bayes, and reinforcement learning (RL) algorithms. To do this, IBM FL integrates with libraries such as Keras, Pytorch, Tensorflow, Scikit-learn, and RLlib. The framework also includes various FL strategies. The FedAvg and iterative average are implemented for NN and RL. In addition to them, PFNM, gradient average, and others can be used for NN. Another difference from other frameworks is the support for several connection protocols, including the Flask web framework, gRPC, and WebSockets.

Both FL libraries are not proprietary and are not distributed under an open license, so they are not discussed in detail in this article.

## 5. Evaluation of Open-Source Federated Learning Frameworks

### 5.1. Experimental Settings

To test the capabilities of each framework, the authors performed a series of experiments with them on the same data sets. Google Cloud virtual machines were used as a runtime environment. The following virtual test beds were created:

- The first one was used to test the simulation mode on one virtual machine; it had the following characteristics: single-hardware hyper-thread in a 2.0–3.8 GHz core, 6.5 GB RAM (RW speed: 24 Mb/s), swap: 180 GB, HDD: 200 GB (RW speed: 24 Mb/s);
- The second one was used to test the federated mode; it consisted of three instances: one server and two clients. The server and the clients had the following characteristics: single-hardware hyper-thread in a 2.0–3.8 GHz core, 6.5 GB RAM (RW speed: 24 Mb/s), swap: 180 GB, HDD: 30 GB (RW speed: 24 Mb/s).

As none of the open-source FL frameworks described above support GPUs, the authors only used CPUs.

The instances’ characteristics were similar in all experiments and for all frameworks. To test the FL frameworks’ capabilities, the authors used three different data sets (Table 4):

- A small signal data set that contains data that describe a car’s parameters during its movement. These parameters are collected using smartphones and include such features as altitude change, vertical and horizontal accelerations, average speed and its variance, engine load, cooling temperature, revolutions per minute (RPM), etc. The data were collected from two vehicles—a Peugeot 207 1.4 HDi (70 CV) and Opel Corsa 1.3 HDi (95 CV). The goal of the data set is to determine the overall state of the system consisting of the car, driver, and road, and therefore, the target labels are the road surface, driving style, and traffic state [50].
- A signal data set that also describes the movement of two vehicles—dumpers of AH and HL series. These dumpers operated at a ground remediation site in Sweden, and the collected attributes include the timestamp, speed, gyroscope, and accelerometer data [51]. The objective of the data set is to define the state of the commercial vehicle—movement, idle, loading, and discharge. The data set is not balanced, as it contains a log of more than five hours from the AH dumper and only a 75-minute log from the HL dumper.
- An image data set that contains X-ray images obtained from 5232 patients of pediatric medical centers and labeled according to two categories: pneumonia and normal [52]. It includes 3883 images with signs of pneumonia and 1349 normal images.

The authors compared the FL frameworks to each other as well as to other popular deep learning libraries: TF and PyTorch. Considering that FATE uses only Dense layers in all frameworks, the authors used NN with the following Dense layers to analyze signal data sets:

- An input layer with the number of nodes corresponding to the number of data set properties;
- An intermediate fully connected layer with 256 nodes;
- An intermediate fully connected layer with 64 nodes;
- An output layer.

NN training was implemented with the following parameters:

- Number of epochs = 20;
- Batch size = 32;
- Number of rounds = 20;
- Stochastic gradient descent (SGD) optimizer;
- Learning step = 0.01.

Obviously, these parameters are not optimal, but they were chosen based on the ability to perform experiments on all frameworks in an acceptable time and with an acceptable quality.

To analyze the image data set, the following structure of convolutional neural networks (CNN) was used:

- An input convolutional layer for a 150 × 150 image;
- Two intermediate convolutional layers with 64 nodes;
- An intermediate convolutional layer with 128 nodes;
- An intermediate convolutional layer with 256 nodes;
- An intermediate Dense layer with 256 nodes;
- An output Dense layer with one node.

CNN training was implemented using the following parameters:

- Number of epochs = 10;
- Batch size = 32;
- Number of rounds = 20;
- SGD optimizer;
- Learning step = 0.001.

In the experiments, the data augmentation was not applied in the image classification task, though it is a common practice to increase the accuracy of the analysis model [53]. In FL, it is also applied to solve the problems with statistical heterogeneity of data due to the collection of data from different devices [54]. However, in major cases, data augmentation is a resource-consuming task in terms of computation, time, and communication, even for solutions that are specially tailored for IoT environments [55,56].

Moreover, the application of data augmentation is often impossible due to security issues, such as the lack of permissions to write data. For these reasons, augmentation was not used in any of the frameworks, including TF and PyTorch, though these frameworks support this technique, and its implementation would improve the accuracy of the trained model.

**Figure 9:**
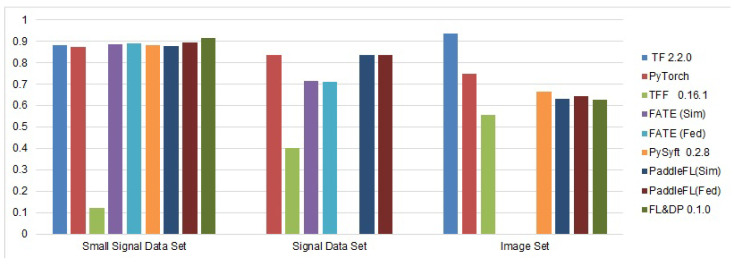
Accuracy of the FL frameworks.

**Figure 10:**
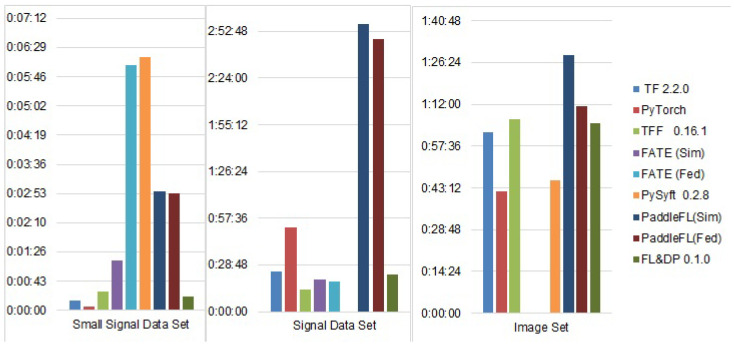
Training time of the FL frameworks.

The results of the experiments are shown in Figure 9 and Figure 10. Figure 9 shows the accuracy of the selected FL frameworks and other ML frameworks on the selected data sets, and Figure 10 shows the time spent by each framework to train the selected model for each data set.

### 5.2. Experimental Results and Discussion

The PFL framework showed the best training accuracy rate for each data set. Its accuracy is comparable to the results obtained using TF and PyTorch. The training accuracy for the small signal data set is almost the same for all frameworks except TFF. For the signal data set, the accuracy of the trained NN on the PFL and FL & DP frameworks is the highest and is 15% higher than FATE’s one. The TFF training accuracy is the lowest one, while PySyft was unable to train the NN, generating an “out of memory” error.

For the image data set, PFL showed the best accuracy of all FL frameworks; however, it is 10% lower than TF’s accuracy and 10% higher than PyTorch’s accuracy. PySyft and FL & DP showed approximately the same results, which are 10% lower than PyTorch’s result. The TFF had the lowest accuracy rate for this data set.

The low accuracy of TFF on all data sets can be explained by the simplified implementation of the aggregation functions (sum and mean), which do not provide a sufficient increase in accuracy when combining models built on a reduced amount of data on each of the clients after splitting the data set.

The accuracy rates of the constructed NNs in the simulation and federated modes in PFL and FATE do not differ. This fact confirms the possibility of using the simulation mode to configure NNs with their further usage in the federated mode without any changes.

All FL frameworks showed longer training times for the small signal data set in comparison with TF and PyTorch. At the same time, for the signal data set, all FL frameworks except PFL had training times less than those of TF and PyTorch. This fact is explained by the time spent on transferring the model between clients and the server and on aggregating parallel-built models. When processing a small dataset, these time costs are significantly higher than the training time and are not compensated by parallel processing of the split data. When processing a large dataset, the training time is reduced by parallel training and thus compensates the time cost of transferring the model between the client and the server.

The PFL constitutes an exception among the FL frameworks reviewed, as it showed significant training times on all data sets, with the longest one being for the signal data set. It should be noted that the training time for the same model on the same set with the same parameters on PaddlePaddle also took more than two hours. Consequently, such a long training time could be explained by the specific backend implemented in PFL. The training time for PFL was the same on all data sets in the simulation and federated modes.

The longest training time on the small data set was shown by PySyft. Considering that training on the signal data set for PySyft took much longer and was unsuccessful, the authors believe that processing of this type of data in the framework is not fully debugged, and this was confirmed on the developer forums.

TFF and FL & DP had relatively short training times. This can be explained by the simple aggregation functions that are used in these frameworks. However, this results in accuracy loss for TFF, while it does not impact FL & DP’s accuracy.

The authors also tried to build a linear regression model and decision tree model for the signal data sets in FATE, PFL, and FL & DP. Unfortunately, it took a very long time for FATE and failed at the end.

The linear model for the signal data set was successfully trained for PFL and FL & DP. Thus, for PFL, the model was built in both the simulation and federated modes. In both modes, the training was successful and gave comparable results. The accuracy expectedly dropped by 30% compared to using an NN. At the same time, the training time was also more than one hour, which is not typical for algorithms of this type.

In the FL & DP framework, we trained linear and logistics models for the signal data set. The accuracy of these models was lower when using an NN, but the training time was significantly shorter.

## 6. Conclusions

We compared all federated learning frameworks and evaluated their characteristics. Currently, all of them are being actively developed, and soon, they are expected to have new features and new properties. However, they already allow performance of federated learning in the simulation mode, which makes it possible to start adopting this technology in industrial systems. On all the frameworks considered, one can start building models based on neural networks, which can be used in production in the next year.

The results of the evaluation of the federated learning frameworks’ features and experiments shows that PFL is the most ready for industrial use. However, it has poor documentation and a small developer community. The disadvantage of this framework is also the use of its own DL PaddlePaddle platform, which is not well known. Moreover, PFL has the longest training time.

The federated mode is also implemented in the FATE framework. It works well for deep learning, but has limitations on the neural network layers used. It also contains implementations of decision trees and linear models, but they do not function yet. The authors believe that, in the near future, these shortcomings will be eliminated and FATE can be fully used in production.

Of course, it is necessary to consider the TensorFlow Federated framework from Google. Its main drawback is that it uses only deep learning techniques, which have several disadvantages, including low explainability and long training time.

The Federated Learning and Differential Privacy framework is very easy to learn and to use. It is integrated with TensorFlow and the SciKit-Learn library, which allows implementation of not only deep learning algorithms, but also other machine learning techniques. So far, it is the only system that provides a solution for clustering tasks.

All of these frameworks are designed for use in cross-silo systems because their installation is quite complicated, and the part allocated to clients requires large memory resources. For this reason, the authors conclude that, currently, the application of these frameworks in the IoT environment is almost impossible, with the exception of the PySyft framework. PySyft is included in the OpenMined ecosystem, which has projects aiming at supporting iOS and Android platforms. Thus, it could be used on mobile devices and, possibly, IoT devices.

## Figures and Tables

**Table 1:** Distributed learning vs. federated learning.

	Distributed Learning	Federated Learning
Aims	Scalable parallelized big data processing	Processing distributed data on heterogeneous data sources
Datasets	IID (identically and independently distributed) and have roughly the same size	Heterogeneous data sets; their sizes may span several orders of magnitude
Nodes	Data centers with powerful computing resources connected by high-performance networks	Often low-power devices, such as smartphones and IoT devices, with limited computing resources connected by less reliable networks, which results in their failures or dropping out of computation

**Table 2:** Properties of FL systems.

Properties of Clients	Cross-Silo	Cross-Device
Computation and storage capacity	High-performance	Low
Scale	Relative small	Large
Stability	High	Low
Data distribution	Usually non-IID	Usually IID

**Table 3:** Comparison of the open-source federated learning systems’ (FLSs’) features.

Features	TFF 0.17.0	FATE 1.4.4/1.5	PySyft 0.2.8	PFL 1.1.0	FL & DP 0.1.0
OS	Mac Linux	Mac Linux	Mac Linux Win iOS Android	Mac Linux Win	Linux Win
Settings	Cross-silo	Cross-silo	Cross-silo Cross-devices	Cross-silo Cross-devices (in future)	Cross-silo
Data Partitioning	Horizontal	Horizontal Vertical	Horizontal Vertical	Horizontal Vertical Transfer	Horizontal
Data type	Time series Images	Time series	Images	Time series Images	Time series Images
Mode	Simulation	Simulation Federated	Simulation Federated	Simulation Federated	Simulation
Clustering model	No	No	No	No	Yes (Kmeans SciKitLearn)
ML Model	No	Yes (very slow)	No	Yes	Yes (SciKitLearn)
Decision Tree Model	No	Yes (very slow)	No	No	No
Protocol	gRPC/proto (in future)	gRPC/proto	Doesn’t use	ZeroMQ	Doesn’t use

**Table 4:** Experiment data sets.

Data Set	Columns	Rows	Volume
*Small signal data set*	13	30,000	3.5 MB
*Signal data set*	9	1.5 million	157 MB
*Image data set*	6000 (Number of images 150 × 150)		1.15 GB

## Abbreviations

The following abbreviations are used in this manuscript:

AFL	Agnostic federated learning
BTSB	Boosting tree secure boost
BYOC	Bring your own components
CCPA	California Consumer Privacy Act
CNN	Convolutional neural network
CP	Computing party
DL	Deep learning
DNN	Deep neural network
DP	Differential privacy
DPSGD	Differentially private stochastic gradient descent
FATE	Federated AI Technology Enabler
FedAvg	Federated averaging
FedMA	Federated matched averaging
FedProx	Federated proximal
FedSGD	Federated stochastic gradient descent
FL	Federated learning
FL & DP	Federated Learning and Differential Privacy
FLS	Federated learning system
GBDT	Gradient-boosting decision tree
GDPR	General Data Protection Regulation
HE	Homomorphic encryption
IBM FL	IBM Federated Learning
IID	Independent and identically distributed
IP	Input party
IoT	Internet of Things
IOWA	Induced ordered weighted averaging
LogR	Logistic regression
LR	Linear regression
LSTM	Long short-term memory
ML	Machine learning
MMARS	Medical Model ARchive
MPC	Multi-party computation
NN	Neural network
OWA	Ordered weighted averaging
PDPA	Personal data protection
PFF	Prefix frequency filtering
PFL	Paddle Federated Learning
PFNM	Probabilistic federated neural matching
PR	Poisson regression
PSI	Private set intersection
RL	Reinforcement learning
RNN	Recurrent neural network
RP	Result party
RPM	Revolutions per minute
SecAgg	Secure aggregation
SGD	Stochastic gradient descent
SPDZ	SecretShare MPC Protocol
TEE	Trusted execution environment
TF	TensorFlow
TFF	TensorFlow Federated
